# A UK national survey of care pathways and support offered to patients receiving revision surgery for prosthetic joint infection in the highest volume NHS orthopaedic centres

**DOI:** 10.1002/msc.1186

**Published:** 2017-03-23

**Authors:** Andrew J. Moore, Michael R. Whitehouse, Rachael Gooberman‐Hill, Jason Heddington, Andrew D. Beswick, Ashley W. Blom, Tim J. Peters

**Affiliations:** ^1^ Musculoskeletal Research Unit University of Bristol UK

**Keywords:** arthroplasty, orthopaedic infections, patient‐centred care, surgery

## Abstract

**Background:**

Deep prosthetic joint infection (PJI) is a devastating complication of joint replacement surgery. It is difficult to treat, and patients often require multiple major revision surgeries to eradicate the infection. Treatment can have negative and long‐term impact on patients' quality of life. Understanding current service provision provides valuable information needed to design and evaluate support interventions for patients.

**Aim:**

This survey aimed to identify usual care pathways and support in UK National Health Service (NHS) orthopaedic centres for patients receiving revision surgery for PJI after hip or knee replacement.

**Methods:**

The 20 highest volume NHS orthopaedic centres treating prosthetic joint infection after hip or knee replacement were approached. Consultant orthopaedic surgeons specializing in treating PJI were invited to participate in a telephone or email survey about usual care provision and support for PJI.

**Findings:**

Sixteen centres completed the survey. Findings showed a high degree of variation nationally in follow‐up time‐points after revision surgery. Multidisciplinary approaches to care focused more on clinical care and physical rehabilitation than social and psychological care. Patient management and referral to support services also varied and barriers to referrals included lack of availability or access to services, lack of knowledge of services, shortage of staff, and complexities of referring outside of the hospital catchment area.

**Conclusions:**

Our findings suggest that future development of interventions should focus on more inclusive and patient‐centred multidisciplinary approaches to care. Such interventions could more completely address psychological and social as well as physical aspects of patients' recovery.

## 1 INTRODUCTION

Approximately 187 000 people have hip or knee replacements in England, Wales, Northern Ireland and the Isle of Man each year (National Joint Registry, 2016a, 2016b). Perhaps the most devastating complication is deep prosthetic joint infection (PJI), which affects about 1% of patients (Blom, Taylor, Pattison, Whitehouse, & Bannister, 2003; Blom et al., 2004) although in some units estimates are as high as 5% (Briggs, 2015). Treatment costs to the National Health Service (NHS) for each patient with PJI are around £100 000 (Briggs, 2015). Infections occurring within 2 years of surgery are usually acquired during the operation, while infections occurring beyond 2 years are predominantly acquired through seeding from blood (Zimmerli, Trampuz, & Ochsner, 2004). Histories of diabetes, rheumatoid arthritis, depression, steroid use and previous joint surgery are associated with an increased risk of developing PJI (Kunutsor, Whitehouse, Blom, Beswick, & Team, 2016). Symptoms include inflammation, severe pain, loss of function, discharge from the surgical wound, fever and nausea; if left untreated, infections can result in disability and death (Hunter & Dandy, 1977; Zimmerli et al., 2004). PJI is difficult to treat, and patients often require multiple major surgeries and antibiotic therapy to eradicate the infection (Moore, Blom, Whitehouse, & Gooberman‐Hill, 2015). PJI is associated with poor quality of life (Cahill, Shadbolt, Scarvell, & Smith, 2008), while re‐infection occurs in around 8% of cases (Kunutsor, Whitehouse, Blom, Beswick, & INFORM Team, 2015; Kunutsor, Whitehouse, Lenguerrand, et al., 2016) and complications associated with surgery are common (Johnson, Sayeed, Naziri, Khanuja, & Mont, 2012; Jung, Schmid, Kelm, Schmitt, & Anagnostakos, 2009).

Patients' experiences of PJI and treatment reveals that the infection itself and treatments impose heavy physical, social and psychological burdens. A study of patients from five high‐volume NHS hospitals who underwent treatment for PJI following hip replacement showed that patients' surgical histories are often complex, extend over many years and often involve multiple surgical revisions in an effort to eradicate infection (Moore et al., 2015). Patients experience sudden and very negative changes in their quality of life, enduring severe pain and long periods of immobility, resulting in an inability to participate in daily personal, work and leisure activities leading to social isolation, and economic difficulties. These changes can persist into the long‐term and some people have life‐long chronic infections (Moore et al., 2015; Andersson, Bergh, Karlsson, & Nilsson, 2010). Psychologically, patients experience anxieties that the infection might not heal or that it may return (approximately 8% experience re‐infection within 2 years) (Beswick et al., 2012; Kunutsor et al., 2015; Kunutsor, Whitehouse, Lenguerrand, et al., 2016), the implications of which are further surgery, long‐term disability and possible amputation. Some patients report depression and suicidal thoughts (Moore et al., 2015; Andersson et al., 2010). Cahill et al. (2008) note that more psychological and social support is needed for this group of patients, due to the negative impact on their social functioning and mental health. Patients rated their overall quality of life as poor with 12% of patients rating their current situation equivalent to, or worse than, death. Socially, the burden of care can impact negatively on members of the patient's family, who often become carers, and while personal relationships can be a source of support they can also come under great strain (Moore et al., 2015; Andersson et al., 2010). Patients also report having to resign from employment, with consequent financial concerns (Moore et al., 2015; Andersson et al., 2010). Some older and less mobile patients report having to move residence, including unplanned downsizing or moving into nursing homes (Moore et al., 2015).

Once discharged home, patients report numerous unmet complex needs that adversely affect their quality of life, including psychological support and physical rehabilitation. Patients describe feeling unprepared for the impact of revision treatment on themselves and their family, while those living alone are vulnerable to social isolation and loneliness. The physical implications of infection and treatment are also linked to distress, concern and uncertainty, with more rehabilitative support needed both during and after treatment and longer‐term recovery (Moore et al., 2015).

PJI is a complex condition that requires multidisciplinary medical and social management over the perioperative period and beyond. In patients with PJI this may range from months to years. Where patients have multiple unmet needs during and beyond the perioperative period it is reasonable to assume that a multidisciplinary approach to care may improve patient outcomes. Such approaches involve clinical and allied healthcare professionals (e.g. dietician, community nurses, orthogeriatricians) working together to formulate and deliver integrated patient‐centred care to address the physical and psychosocial needs of the patient in an attempt to improve the patient's journey through clear communication, leadership, collaboration, and the streamlining of diagnostics and therapeutics (Borras et al., 2014; Denton & Conron, 2016).

Currently, there are no national guidelines or Department of Health advice on how best to manage patients with PJI. This may in part be due to a lack of consensus about the best way to treat PJI (Kunutsor et al., 2015; Kunutsor, Whitehouse, Lenguerrand, et al., 2016; Wongworawat, 2013). The current provision of NHS orthopaedic services is varied (Briggs, 2012) and it is uncertain what services are currently provided for PJI patients and how they are provided. Briggs (2012) suggests that there are large disparities in orthopaedic care and referrals in the NHS, and highlights the need for changes to improve pathways of care within these services, to improve patient experiences and outcomes. The report recommends that PJI is treated in specialist orthopaedic hospitals and that enhanced recovery pathways are developed – ensuring that patients receive the right treatment in the right place at the right time, with appropriate follow‐up for patients (Briggs, 2012).

Our previous work has documented the impact of PJI on patients and unmet needs during and after surgery (Moore et al., 2015). Combined with the large disparities in care (Briggs, 2015), management and care of patients with PJI should be a key research and clinical priority, starting with an exploration of current care pathways and referral processes. Findings from our previous research (Moore et al., 2015) and engagement with patients (Gooberman‐Hill et al., 2013) indicates a pressing need to explore: what kind of support is available to patients with PJI; how they are managed during the perioperative period; whether they are referred to other services; and what clinical follow‐up they receive.

The aim of this study is to ascertain the scope of current service provision for patients treated for PJI after hip or knee replacement, and to identify potential barriers and facilitators to care provision for this population within NHS orthopaedic treatment centres that specialize in and deliver treatment for PJI.

## 2 METHODS

High‐volume NHS orthopaedic centres, defined as those that perform more than 500 hip or knee operations per year, were identified from the National Joint Registry, which collects information on joint replacement procedures performed within the NHS (Judge, Chard, Learmonth, & Dieppe, 2006; National Joint Registry, 2016c). The 20 highest‐volume orthopaedic centres treating PJI after hip or knee replacement in 2014 were identified. The orthopaedic department was contacted by telephone by a member of the research team and an appropriate consultant orthopaedic surgeon specializing in PJI was identified. This individual was then contacted by email inviting them to participate in the survey by telephone or email. Data were collected for the survey between November 2015 and September 2016.

The project was conducted as a service evaluation with agreement from the local NHS Trust and the NHS Research Ethics Committee (REC) that formal ethical approval was not required.

## 3 SURVEY QUESTIONNAIRE

A structured survey questionnaire was developed in collaboration with an infection‐specific Patient and Public Involvement group, which is part of the Patient Experience Partnership in Research (PEP‐R) group (Gooberman‐Hill et al., 2013). We based this process on procedures established in previous studies of service provision for post‐operative total hip replacement and total knee replacement patients (Artz et al., 2013; Wylde, Mackichan, Dixon, & Gooberman‐Hill, 2014). The questionnaire was designed to ascertain service provision and care pathways for patients receiving surgical revision treatment for PJI after hip or knee replacement. The questionnaire covered topics including: follow‐up times after revision surgery; communication pathways in the event of patient concerns or complications; routinely used outcome measures; type of support patients may be referred to; barriers to referrals; existence of specialist PJI clinics; and multidisciplinary approaches to care.

## 4 DATA ANALYSIS

Participants' answers to the telephone questionnaire were recorded on a standardized pro forma by the researcher. Telephone and emailed questionnaires were then saved to an encrypted database on a secure server. Questionnaire responses were anonymized through the allocation of an identification number for each centre. Responses were then exported into Microsoft Excel (Microsoft Office 2013) where they were reviewed and frequency statistics generated. Data summaries were then developed for each survey question and reviewed to ensure they reflected the raw data. Finally, a descriptive summary of the complete data set was developed.

## 5 RESULTS

Respondents from 16 of the 20 highest volume orthopaedic centres completed the survey. In the year 2014, these 20 NHS orthopaedic centres accounted for 633 cases treated for PJI of the hip or knee. The 16 centres that responded account for 560 revision cases (88%) of the total of 633 cases. The distribution of these orthopaedic centres included North West (*n* = 1), West Midlands (*n* = 2), East Midlands (*n* = 2), South West (*n* = 3), South East (*n* = 5), East of England (*n* = 1), Yorkshire and Humber (*n* = 1) and South Wales (*n* = 1). All respondents were consultant orthopaedic surgeons. Eleven respondents opted for the email questionnaire, and five respondents opted to complete the questionnaire by telephone. Time to complete the questionnaire was approximately 10–15 min.

### 5.1 Follow‐up of patients who have received revision surgery for PJI

The time‐points at which patients were routinely followed up after revision for infection varied across centres. Most centres saw patients at 6 weeks after revision surgery (*n* = 10). Other centres followed up patients at 2 weeks (*n* = 3), weekly (*n* = 2) or within 4 weeks (*n* = 1) if the infection was still present. Longer‐term follow‐up also varied, with centres following up at 3, 6 and 12 months. Nine centres stated that follow‐up time‐points were standardized (although there was some variation dependent upon the patient's needs). Seven centres stated that follow‐up time‐points varied between consultant orthopaedic surgeons. One centre reported that they were trying to standardize their process.

After being discharged following revision for infection, the period that patients were followed‐up varied between centres and consultants. Centres follow up patients annually indefinitely (*n* = 10), for up to 5 years (*n* = 2) or 10 years (*n* = 1), until the infection cleared (*n* = 1), or up to 3 months if the infection has cleared (*n* = 1).

In the majority of centres (*n* = 15) a consultant conducted the follow‐up appointment. Commonly a registrar would be present (*n* = 10), and in six centres a microbiologist was also present. In other centres an extended scope practitioner (*n* = 5), a clinical nurse specialist (*n* = 1) or nurse practitioner (*n* = 1) would also attend. One centre stated that follow‐up was conducted by the ‘bone infection team’ with a specialist registrar present, and sometimes a consultant. It was established that only a small number of centres (*n* = 4) held a dedicated PJI clinic. Twelve centres stated they had no infection clinic.

Advice for patients on who to contact if they had any concerns varied greatly. The four units with a dedicated infection unit asked patients to call the infection clinic or Outpatient Parenteral Antimicrobial Therapy service. Seven centres asked patients to phone the consultant or the consultant's secretary. One centre asked patients to liaise with a community nurse, consultant's secretary, general practitioner (GP) or Emergency Department. One centre had a dedicated orthopaedic community service that would see patients the same day. One centre gave out advice leaflets, although no further detail about contacts was given, and one centre gave out a card with contact numbers. Two centres discouraged patients from contacting their GP in the first instance.

Making an appointment to be reviewed varied considerably between centres and most centres had no named person or clinician. A high proportion was via the secretary, GP or Emergency Department. One hospital gave patients a card with contact details but commented that patients sometimes found it very difficult to make contact with anyone at the hospital or to arrange an appointment to be reviewed. One hospital stated that it was difficult for patients to arrange a review if they lived out of the area.

Nine of 16 centres used the Oxford Hip Score or Oxford Knee Score and four of these centres used these outcome measures in conjunction with the EuroQol EQ‐5D‐5 L. One centre used the Harris Hip Score alongside the Oxford Hip Score, and one centre used the University of California Los Angeles (UCLA) score alongside the Oxford score. Seven centres did not use any standardized outcome measures.

The Oxford Hip Score and the Oxford Knee Score are short, self‐administered questionnaires, which have been validated for use in total hip or total knee replacements. They consist of 12 questions specifically designed and developed to assess pain and function after hip or knee replacement (Dawson, Fitzpatrick, Carr, & Murray, 1996; Dawson, Fitzpatrick, Murray, & Carr, 1998). The Harris Hip Score is commonly used to assess disease‐specific pain and function in total hip arthroplasty patients. Patients are scored on a 0–100 scale based on the degree of pain, function and range of motion (Harris, 1969). The EuroQol EQ‐5D‐5 L is a validated quality of life measure, consisting of a descriptive system (five dimensions; each dimension having five levels) and a visual analogue scale (patient's self‐rated health recorded on a 20‐cm scale) (Brooks & EuroQol Group, 1996). The UCLA score is a ten‐point activity‐level rating, consisting of ten descriptive activity levels ranging from wholly inactive (level 1), to moderate activities such as unlimited housework and shopping (level 6), to regular participation in impact sports such as jogging or tennis (level 10) (Zahiri, Schmalzried, Szuszczewicz, & Amstutz, 1998).

### 5.2 Management of patients with PJI and referral to other supportive services

Five centres suggested those who had a two‐stage revision generally needed more social support, physiotherapy and occupational therapy, or may need respite care or placement in a cottage hospital as they often needed inpatient care for longer than patients who had a one‐stage revision. However, one centre stated that there was little difference between levels of support for one‐stage or two‐stage revision, due to the type of spacer used that allowed for better functional mobility and weight‐bearing. Another centre stated that they use this technique but did not comment on differences in the level of support required. Four centres stated that levels of support would vary depending on the patient's needs rather than being decided *a priori* on the basis of revision type.

Inpatient support and management services for patients being treated for PJI in all centres included physiotherapy and occupational therapy. Fewer than half of centres included social services (*n* = 7), and three centres included counselling. One centre stated they provided ‘dedicated physio[therapy] and occupational therapy on a separate infection ward’ while two centres reported that they provided ‘standard’ or ‘usual’ physiotherapy and occupational therapy. This suggests that there may be a lack of individualization of physiotherapy and occupational therapy services for patients having revision surgery. Once patients were discharged home, most centres stated that they provided patients with physiotherapy (*n* = 15), with some also stating occupational therapy (*n* = 11) and social services (*n* = 7). One centre provided a discharge liaison nurse to plan additional help, and one centre provided ‘hospital at home’ to manage intravenous antibiotics. One centre commented that community services were ‘a waste of time’ as they offer only limited treatment.

During the interim period between a first‐ and second‐stage revision, support and management options provided included physiotherapy and occupational therapy (*n* = 10). At three centres, hospital at home or community services could help with intravenous antibiotics if needed. At three centres, support and management options would vary according to the patients' needs. One centre reported that patients may go to intermediate care for rehabilitation. One centre had no planned support at this stage. Two centres did not answer this question.

Across the centres, referrals to other services were made by consultants, trainees, GPs, ward staff, physiotherapists and extended scope practitioners. There was great variation between centres. One centre stated that it had implemented a special discharge team of nurses collaborating with ward doctors to work out what care and referrals were needed. One centre had a discharge liaison nurse. Two centres reported discussing referrals during multidisciplinary team meetings.

### 5.3 Barriers to referrals

A number of barriers to referring patients to support services were identified across the centres. The most common barrier was a lack of service provision (no service or staff) and difficulties in accessing services (*n* = 11). Five centres reported difficulties in referring patients outside their geographical catchment area and difficulties in financial repatriation. Delays in arranging services (*n* = 4) and a lack of knowledge of what services were available (*n* = 3) were also barriers. Other respondents noted delays in rehabilitation and that poor provision of social services presented barriers, while ‘consultant pride’ could also be a barrier to referrals, suggesting that some consultants may find it unacceptable to refer patients on if they believe it will reflect negatively upon their own performance. One centre also expressed their frustration about dietetics services suggesting that more input was needed as patients were ‘malnourished’ and that ‘wound healing is slow’.

### 5.4 Multidisciplinary care approaches

Multidisciplinary team meetings are a core component of the NHS's drive towards integrated care for long‐term conditions. Eleven centres stated that they had multidisciplinary care plans in place for patients. Five centres stated they had no multidisciplinary care plan for patients with PJI. Input into multidisciplinary care plans varied across centres and could include ‘the infection team’; ‘microbiologists, radiologists and surgeons but not therapies’; consultant orthopaedic surgeon and microbiologist; consultant orthopaedic surgeon and nurse practitioners; and an orthogeriatrician.

Fourteen out of 16 centres reported that they have multidisciplinary team meetings. Two centres stated they did not have multidisciplinary team meetings. Frequency of meetings varied from once a week to every 2 months. Two centres said the surgeon/consultant did not get involved in the meetings.

## 6 DISCUSSION

This survey of service provision for PJI after total hip or knee replacement found a high degree of variation nationally in follow‐up processes, patient management and referral to support services, and multidisciplinary approaches to care. The majority of centres reported a standard follow‐up time‐point of 6 weeks, similar to the standard orthopaedic follow‐up for treatment‐based decisions for postoperative recovery and bone healing, while some centres quoted earlier follow‐up time‐points such as 2 weeks, which equates to a clinical review to, for example, check wound healing or antibiotic treatment. In over half of the centres that responded, follow‐up time‐points were standardized, while at other centres time‐points varied between surgeons, although one centre suggested they were trying to standardize follow‐up time‐points. The benefit of standardizing follow‐up time‐points is that patients will know in advance when they are being reviewed and will be able to plan visits to the hospital, which may be of psychological benefit as well as convenience to the patient. There are also financial implications as clinical reviews cost around £112.50 per patient (Department of Health, 2016). For longer‐term follow‐up, 10 of the 16 centres reviewed patients annually indefinitely. Other centres limited follow‐up to 5 or 10 years, or until the infection had cleared. Following PJI, the risk of infection recurrence within 2 years is around 8% (Kunutsor et al., 2015; Triantafyllopoulos et al., 2017) and revision for other causes is also high with the 10‐year risk of re‐revision for hips at 15% and 12‐year risk for knees at 17% (National Joint Registry, 2016d). We suggest that as the risk of re‐revision in this population is high, standardizing follow‐up time‐points and indefinite follow‐up would be judicious.

For the majority of centres, follow‐up appointments are conducted by a consultant orthopaedic surgeon and microbiologist and in seven centres an extended scope practitioner (e.g. physiotherapist), clinical nurse specialist or nurse practitioner is also present. Given that our previous research and that of others shows that PJI has a long‐term impact and a considerable number of unmet needs (Moore et al., 2015; Andersson et al., 2010), the inclusion of a multidisciplinary team during follow‐up appointments may offer the opportunity for an earlier and more holistic assessment of the patient's care needs, with signposting of local support services and other therapies. Although 14 centres reported that they had multidisciplinary team meetings, the frequency of these varied greatly between centres. Eleven centres had multidisciplinary care plans, although input was generally from the orthopaedic/surgical team, which often included a microbiologist, rather than from other clinical areas such as physiotherapy or social services. Apart from one centre, which involved an orthogeriatrician, our findings show there was little representation of other clinical and allied healthcare professionals during follow‐up or providing input to multidisciplinary care plans. We suggest there is not an inclusive multidisciplinary approach to the care of patients treated for PJI, and there may be missed opportunities for other clinicians and allied healthcare professionals to be involved perioperatively and in the longer term. Just over half of the centres that responded did not use any standard outcome measures for PJI patients, which suggests there may be a lack of comparable clinical outcome data on changes in patients' pain, function or psychological status, or quality of life in the years since contracting an infection. This important information may be useful to other members of the multidisciplinary teams involved in the rehabilitation of patients, such as physiotherapists, occupational therapists or counsellors, as a way of recording improvements or deterioration over time.

Referrals to other services mostly included physiotherapy and occupational therapy. Fewer than half of centres included social services and only three centres referred patients to counselling services. Research on the impact of PJI (Moore et al., 2015; Andersson et al., 2010; Barrack, Engh, Rorabeck, Sawhney, & Woolfrey, 2000; Cahill et al., 2008) shows that although there is a heavy physical burden on patients, the social and psychological impact of infection and treatment can also be devastating, and the results of this survey suggest these aspects are less supported by current care and management pathways.

Surgeons in this survey identified a number of barriers to onward referrals including lack of service provision and access to some services, and problems referring patients from outside of the local NHS Trust catchment area. Given the recommendation for tertiary referral treatment for patients with PJI, the identified problem of referral for ongoing support following complex and costly surgical intervention is of particular concern. A lack of knowledge of available services was also identified by three centres, which raises further questions about the added value of having a more multidisciplinary approach to the management of PJI, where other more generalized specialities such as orthogeriatricians may be better placed to offer a more holistic and person‐centred approach (British Geriatrics Society, 2007). It is not unreasonable to compare the multifaceted impact of PJI with that of other conditions such as cancer, as both share uncertain outcomes for radical surgical treatment, anxiety associated with the possible return of a malignant condition months or years later, and the heavy physical and psychological burden that the condition and its treatment impose on patients and their family (Simard et al., 2013). In cancer care a person‐centred, holistic and multidisciplinary approach is the standard (Gysels, Higginson, Rajasekaran, Davies, & Harding, 2004), and it may be that future collaboration between specialists from both areas may help to improve services for PJI.

## 7 STRENGTHS AND WEAKNESSES

Although not all the centres replied to our survey, the 16 we surveyed accounted for 88% of the revisions for PJI across the 20 centres. Views on support and management of PJI were supplied by consultant orthopaedic surgeons and although they specialize in the surgical treatment of PJI, it may be that other practitioners, such as nurses or extended scope practitioner physiotherapists, would have different perspectives on patient care and management, particularly in the post‐operative stages. Although this may be a limitation of the survey, participants were given scope to explain their answers. Another limitation of this survey method is that consultant surgeons' perceptions of service provision in their unit may not reflect actual practice, and other surgeons and clinicians in the same unit may have a different experience of what occurs in practice. We aim to conduct further research into care pathways for PJI using qualitative methods, which will help us to gain further understanding of service provision for PJI.

Caution should be used when interpreting these results as we did not set out to suggest whether differences in service provision were associated with differences in clinical outcomes. What we have established is that there is a large variation between a group of high‐volume orthopaedic treatment centres in terms of follow‐up, referral and multidisciplinary approaches to care.

## 8 CONCLUSIONS

Although the findings of our evaluation are limited to 16 high‐volume NHS centres, they can be used in the development of research and the implementation of research findings into clinical practice. Our findings suggest that interventions should focus on more inclusive and patient‐centred multidisciplinary approaches to care, which fully address the psychological and social as well as physical aspects of patients' recovery, and their return to full potential. The next steps will be to develop, implement and evaluate enhanced care pathways for people with PJI after hip and knee replacement.
